# Association between attendance at outpatient follow-up appointments and blood pressure control among patients with hypertension

**DOI:** 10.1186/s12872-020-01741-5

**Published:** 2020-10-21

**Authors:** Sajid Mahmood, Zahraa Jalal, Muhammad Abdul Hadi, Kifayat Ullah Shah

**Affiliations:** 1 Department of Pharmacy, Quaid-E-Azam University, Islamabad, 45320 Pakistan; 2 School of Pharmacy, Institute of Clinical Sciences, University of Birmingham, Edgbaston, Birmingham, B15 2TT UK

**Keywords:** Cross sectional study, Blood pressure, Pakistan, Medication adherence, Primary care, Treatment follow-up

## Abstract

**Objective:**

The aim of this study was to assess the impact of regularity in treatment follow-up appointments on treatment outcomes among hypertensive patients attending different healthcare settings in Islamabad, Pakistan. Additionally, factors associated with regularity in treatment follow-up were also identified.

**Methods:**

A cross-sectional study was undertaken in selected primary, secondary and tertiary healthcare settings between September, 2017 and December, 2018 in Islamabad, Pakistan. A structured data collection form was used to gather sociodemographic and clinical data of recruited patients. Binary logistic regression analyses were undertaken to determine association between regularity in treatment follow-up appointments and blood pressure control and to determine covariates significantly associated with regularity in treatment follow-up appointments.

**Results:**

A total of 662 patients with hypertension participated in the study. More than half 346 (52%) of the patients were females. The mean age of participants was 54 ± 12 years. Only 274 (41%) patients regularly attended treatment follow-up appointments. Regression analysis found that regular treatment follow-up was an independent predictor of controlled blood pressure (OR 1.561 [95% CI 1.102–2.211; *P* = 0.024]). Gender (OR 1.720 [95% CI 1.259–2.350; *P* = 0.001]), age (OR 1.462 [CI 95%:1.059–2.020; *P* = 0.021]), higher education (OR 1.7 [95% CI 1.041–2.778; *P* = 0.034]), entitlement to free medical care (OR 3.166 [95% CI 2.284–4.388; *P* = 0.0001]), treatment duration (OR 1.788 [95% CI 1.288–2.483; *P* = 0.001]), number of medications (OR 1.585 [95% CI 1.259–1.996; *P* = 0.0001]), presence of co-morbidity (OR 3.214 [95% CI 2.248–4.593; *P* = 0.0001]) and medication adherence (OR 6.231 [95% CI 4.264–9.106; *P* = 0.0001]) were significantly associated with regularity in treatment follow-up appointments.

**Conclusion:**

Attendance at follow-up visits was alarmingly low among patients with hypertension in Pakistan which may explain poor treatment outcomes in patients. Evidence-based targeted interventions should be developed and implemented, considering local needs, to improve attendance at treatment follow-up appointments.

## Introduction

According to the World Health Organisation report, hypertension is the leading cause of morbidity and mortality in the world and is responsible for nine million deaths every year [1]. Despite the availability of different treatment options, the rate of blood pressure control remains suboptimal [2, 3]. In the United States of America alone, the cost associated with uncontrolled hypertension accounts for $131 billion annually [4]. Patients with hypertension, including those with controlled blood pressure, are twice as likely to develop cardiovascular complications as compared to the patients without hypertension [5]. The risk of stroke and ischemic cardiac events can be reduced to one third if the systolic blood pressure is controlled below 140 mmHg [6]. Effective control of blood pressure can only be achieved through lifelong care and regular follow-up [7, 8]. Regular treatment follow-up is an important component of effective disease management especially for long term conditions [9, 10]. For patients with hypertension, treatment follow-up provides an opportunity for healthcare practitioners to adjust patient’s treatment regimen, assess patient’s adherence to the therapy, monitor any adverse effects and improve patient’s understanding of disease management [11–13]. The research has demonstrated that regular attendance at treatment follow-up appointments is associated with better treatment outcomes in patients with hypertension [8, 9, 14–16]. Several interventions have been tested to improve follow-up care among patients with chronic diseases. These interventions include increased awareness among patients regarding the benefits of follow-up care, providing free medications, free transport vouchers, telephone/sms reminders and appointment assistance. All these interventions have shown positive effect on regularity in follow-up care among patients [12, 17–19].

Although there is slight variation in recommendations for scheduling of treatment follow-up appointments among various hypertension treatment guidelines, all guidelines strongly recommend regular follow-up appointments to monitor treatment outcomes. The European Society of Hypertension (ESH) guidelines for management of arterial hypertension recommends frequent, at least once a month visit to a specialized healthcare facility until the optimal target blood pressure (BP) is achieved. Once the target BP is achieved a visit interval of few months is considered reasonable [20]. The American Heart Association (AHA) 2017 guidelines for prevention, detection, evaluation and management of high blood pressure in adults also recommend scheduling follow-up evaluation at monthly interval until target BP is achieved and should be reassessed every three to six months [21].

According to a recently published meta-analysis, the prevalence of hypertension in Pakistan was 26.4% [22]. The national health survey of Pakistan reported that only 50% of patients with hypertension in Pakistan were diagnosed and of those who were diagnosed, only 50% had ever received any treatment for hypertension [23]. Similarly the control rate of blood pressure was only 12.5% [23]. Both pharmacological and non-pharmacological interventions are required for optimal management of hypertension [24]. Despite of its importance, no data is available on the regularity of follow-up appointments, predictors of regularity in follow-up appointments and association between regular follow-up visits and blood pressure control among patients with hypertension in Pakistan. The purpose of this study was to assess the regularity in hospital follow-up appointments, determining factors predicting regularity in hospital follow-up visits and to find out the impact of regular treatment follow-up visits on blood pressure control among hypertensive patients in Pakistan.. This paper is part of larger study evaluating the predictors of blood pressure control in Pakistan [3].

## Material and methods

### Ethics statement

The ethics approvals of the study were obtained from Bioethics committees/administrations of Quaid-i-Azam University, Islamabad, Pakistan, Pakistan Institute of Medical Sciences Islamabad (tertiary care hospital), Government CDA Hospital, Sector G-6/2 Islamabad (secondary care hospital) and Government CDA Medical Centre, Sector I-10 Islamabad (primary healthcare setting) (Reference number. BFC-FBS-QAU-2018–108,Dated: 23/10/2018, F.1–1/2015/ERB/SZABMU Dated: 28/08/2017 and CDA/DHS-14(1) (63)/2018/1077 Dated: 09/10/2018). Informed written consent was obtained from each participant before enrolment in the study. The anonymity and confidentiality of the survey was guaranteed to each enrolled participant.

### Study settings

The study was conducted in three different healthcare settings in Islamabad, the federal capital of Pakistan. These healthcare settings included one tertiary care hospital, one secondary care hospital and one primary healthcare clinic to allow better generalization of study findings.

### Sample size calculation

The sample size was calculated using an online sample size calculator, Raosoft®. The overall minimum sample size was calculated to be 385 based on 95% confidence interval, 5% margin of error and 50% response distribution. We aimed to recruit at least 100 patients from each study center. The respondents were sampled consecutively from all study centers.

### Study design and participants

**Fig. 1:**
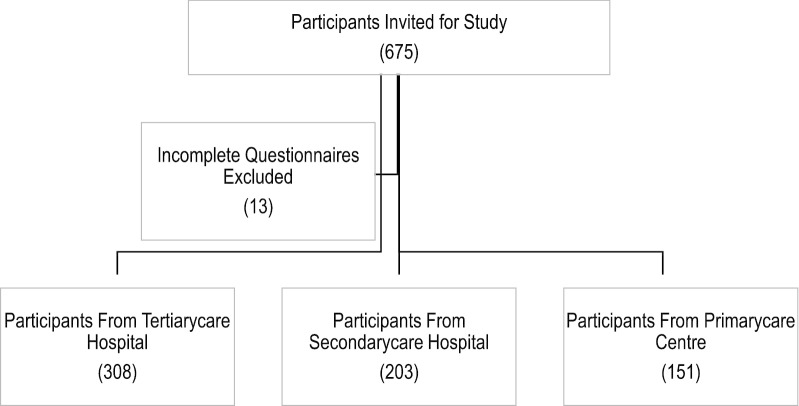
Participant recruitment flow diagram

A cross-sectional methodological approach was adopted. Three hundred and eight patients were recruited from tertiary care hospital, 203 patients from secondary care hospital and 151 patients from primary health care Centre (Fig.1). The participants were recruited from September, 2017 to December, 2018. All adult patients aged 18 and above, diagnosed with essential hypertension, using at least one antihypertensive medication and able to communicate in Urdu (Pakistan’s National Language) were invited to participate in the study. Patient’s hand-held record was used to ascertain their eligibility to participate in the study. Hypertensive patients with comorbidities were also included in the study. Pregnant women, patients with mental health illnesses affecting their cognitive abilities (e.g. dementia, Parkinson’s disease) and those who could not communicate in Urdu language were excluded from our study. Participation in the study was voluntary. Participants meeting the inclusion and exclusion criteria were recruited using consecutive sampling strategy from each of the participating centres.

### Data collection

A structured self-administered and self-reported questionnaire was used to assess the regularity in treatment follow-up appointments. However, for illiterate participants the questionnaires were administered by the interviewer. The data collection was divided into three steps. In the first step, a standard questionnaire was used to collect sociodemographic data of the patient. In the second step, patient’s medical record was reviewed and relevant clinical data were extracted including status of blood pressure control, number/nature of co-morbidities, medication history, risk factors and follow-up schedule advised to the patient. The follow-up schedule advised to the patient was used for the assessment of treatment follow-up regularity. In the third step, the participants were asked following questions relating to the regularity in treatment follow-up visits: When did you visit your doctor last time for treatment follow-up? Do you think that you visit your doctor regularly for treatment follow-up? After how many days/weeks/months do you visit your doctor for treatment follow-up? In last ten scheduled/advised visits, how many times have you missed your follow-up visit?. Eight items Morisky medication adherence scale (MMAS-8) questionnaire was used to assess medication adherence [25–28]. The participants with MMAS-8 score ≤ 6 were considered as adherent to their prescribed antihypertensive therapy.

### Outcome measures and covariates

The primary outcome measure was regularity in follow-up visits. A participant was considered as irregular in treatment follow-up visits if he/she had missed more than three out of 10 scheduled/advised follow-up visits [29–31]. The patients with hypertension were defined in accordance with the National Institute for Health and Care Excellence (NICE) treatment guidelines, 2011 for the management of hypertension. A blood pressure reading of 140/90 and 150/90 was considered controlled for patients aged less than 80 years and more than 80 years respectively. Similarly, for the patients with kidney failure, eye or cerebrovascular damage the blood pressure level under 130/80 mmHg was considered as controlled [32]. The covariates were age, marital status gender, education level, profession, entitlement status,smoking status, number of medications prescribed, duration of therapy, presence of comorbidities and medication adherence.

### Statistical analysis

Statistical Package for Social Sciences (SPSS) version 21.0 was used to perform statistical analysis. A P-value of less than 0.05 was considered statistically significant. The binary logistic regression analysis using Forward Likelihood Ratio method was used to identify predictors associated with regularity in treatment follow-up and blood pressure control. Correlation and Hosmer–Lemeshow Goodness of Fit test was performed to select best prediction model.

## Results

A total of 662 patients with hypertension participated in the study. Of 662 patients, 315 (48%) were male and 346 (52%) were female. The mean (± S.D) age of participants was 54 (± 12) years. The mean (± S.D) duration of hypertension was 6 (± 6) years. The mean (± S.D) systolic blood pressure of participants was 148 mmHg (± 18) and mean (± S.D) diastolic blood pressure was 92 mmHg (± 11). The mean number of antihypertensive drugs used was 1.69 ± 0.7. Two hundred and sixty (39%) participants were obese. Two hundred and eighty-nine (44%) participants were using single antihypertensive agent and, 253 (38%) participants were entitled to free medical care. Two hundred and ninety (44%) participants had at least one co-morbidity. The prevalence of diabetes among participants was 15% 8% of participants had coronary artery disease [CAD], 1% patients had congestive heart failure [CHF] and 21% of participants had hyperlipidemia. The participants’ demographic characteristics are presented in Table 1. Less than half of the patients (41%) were regular in attending follow-up visits. Similarly, in terms of healthcare setting, the number of patients who were regular in attending their follow-up appointments in tertiary care, secondary care and primary care setting were 118 (38%), 123 (61%) and 33 (22%) respectively. (Table 2).

**Table 1:** Demographic and clinical characteristics of recruited patients

Patient characteristics		Total study population (N = 662)	
		N	%
Gender	Male	315	48
	Female	346	52
Age	Mean (SD)	54 ± 12.3 years	
	≥ 60	231	35
	˂60	431	65
Marital status	Married	574	87
	Unmarried/divorced/widow	88	13
BMI	Overweight	260	39
	Normal weight	401	61
Education level	Graduate and above	98	15
	Secondary and higher secondary	228	34
	Primary and below	149	23
	Uneducated	187	28
Profession	Officers/higher management	63	10
	Clerical staff	52	08
	Workers/laborers	122	18
	Self employed	15	02
	Retired/unemployed	115	17
	House wives	295	45
Entitlement status	Entitled	253	38
	Non-entitled	409	62
Treatment duration	Mean (SD)	06 ± 5.66 Years	
	≤ 5 years	442	67
	> 5 years	220	33
Number of antihypertensive drugs	Mean (SD)	1.69 ± 0.681	
	1	289	44
	2	293	44
	3	79	12
Co-morbidity	Yes	177	27
	No	485	73
Specific co-morbidity	Patients with DM	96	15
	Patients with CHD	53	08
	Patients with CHF	05	0.8
	Patients with Hyperlipidemia	136	21
Regularity in follow-up	Regular Follow-up	274	41
	Irregular Follow-up	388	59

**Table 2:** Regularity in treatment follow-up Appointments among patients with hypertension stratified by healthcare setting, entitlement to free medical care and blood pressure control

	No. of patients n	Regular in treatment follow-up appointments n (%)	Irregular in treatment follow-up appointments n (%)
Healthcare setting			
Tertiary care hospital	308	118 [38]	190 [62]
Secondary care hospital	203	123 [61]	80 [39]
Primary healthcare centre	151	33 [22]	118 (78)
Overall	662	274 [41]	388 [59]
Entitlement to free medical care			
Treatment follow-up appointments regularity in entitled patients	258	150 [58]	108 [42]
Treatment follow-up appointments regularity in non-entitled patients	404	124 [31]	280 (69)

Out of 662 participants, 253 patients were entitled to free medical care. Of the patients who were entitled to free medical care, 150 (58%) participants regularly attended treatment follow-up appointments. Whereas the patients who were not entitled to free medical care, only 124 (31%) participants attended regular treatment follow-up appointments (*P* = 0.0001) (Table 2).

The results of binary regression analysis found that gender, age, higher education, entitlement status, treatment duration, number of medications, presence of co-morbidity, medication adherence and blood pressure control were significantly associated with regularity in treatment follow-up appointments. Similarly marital status, body mass index (BMI) and profession had no significant association with regularity in treatment follow-up visits. (Table 3).

**Table 3:** Factors predicting regularity in treatment follow-up appointments

Parameter	Irregular in treatment follow-up n (%)	Regular in treatment follow-up n (%)	OR	95% CI for OR lower–upper	*P* value
Gender					
Female	225 (65)	122 [35]	1		
Male	163 [52]	152 (48)	1.720	1.259–2.350	0.001*
Age					
< 60 Years	266 (62)	164 (38)	1		
≥ 60 Years	121 (52)	110 (48)	1.462	1.059–2.020	0.021*
Marital status					
Married	342 (60)	232 (40)	1		
Unmarried/divorced/Widowed	46 (52)	42 (48)	1.346	0.858–2.111	0.196
Education					0.166
Uneducated	116 (62)	71 (38)	1		
Primary or below	86 (58)	63 (42)	1.197	0.771–1.857	0.423
Secondary and higher secondary	137 (61)	89 (39)	1.061	0.713–1.580	0.769
Graduation and above	49 (49)	51 (51)	1.700	1.041–2.778	0.034*
BMI					
Normal weight	237 (59)	164 (41)	1		
Obese	151 (58)	110 (42)	1.053	0.767–1.444	0.750
Profession					0.384
Retired/unemployed/house wives	248 (60)	162 (40)	1		
Officers	32 (51)	31 (49)	1.483	0.871–2.525	0.147
Clerical staff	26 (50)	26 (50)	1.531	0.858–2.730	0.149
Worker/laborer	67 (55)	55 (45)	1.257	0.836–1.890	0.272
Self employed	15 (100)	0 (0)	-	-	-
Entitlement status					
Non-entitled	283 (69)	126 (31)	1		
Entitled	105 (42)	148 (58)	3.166	2.284–4.388	0.0001^*^
Treatment duration					
≤ 5 years	281 (63)	163 (37)	1		
> 5 years	107 (49)	111 (51)	1.788	1.288–2.483	0.001^*^
No. of medications					
1	199 (69)	90 (31)			
2	147 (50)	146 (50)			
3	41 (52)	38 (48)	1.585	1.259–1.996	0.0001^*^
Co-morbidities					
No	321 (66)	164 (34)	1		
Yes	67 (38)	110 (62)	3.214	2.248–4.593	0.0001^*^
Medication adherence					
Non-Adherent	211 (83)	44 (17)	1		
Adherent	177 (44)	230 (56)	6.231	4.264–9.106	0.0001^*^

Significant values are highlighted in bold

*P < 0.05

The level of adherence was measured through 8-items Morisky Medication Adherence Scale (MMAS-8). Use of the MMAS is protected by US copyright laws. Permission for use is required. A license agreement is available from: Donald E. Morisky, ScD, ScM, MSPH. Use of the ©MMAS is protected by US copyright and registered trademark laws. Permission for use is required. A license agreement is available from: Donald E. Morisky, 294 Lindura Court, Las Vegas, NV 89,138–4632; dmorisky@gmail.com.The scale's questions are available in the originally published article (25)

For factors associated with treatment follow-up regularity, the results of binary regression analysis showed that males were 1.7 times more likely to regularly attend follow-up meetings compared to females (OR 1.720 [95% CI 1.259–2.3]). The participants aged ≥ 60 years of age were 1.5 times more likely to be regular in treatment follow-up visits than participants who were under 60 years of age (OR 1.462 [95% CI 1.059–2.020]). The participants who were entitled to free medical care were approximately three times more likely to attend follow-up appointments regularly compared with patients who were not entitled to free medical care (OR 3.166 [95% CI 2.284–4.388]). Patients with a co-morbidity were 3.2 times more likely to be regular in treatment follow-up visits than the patients without any co-morbidity (OR 3.214 [95% CI 2.248–4.593]). Patients with good medication adherence were approximately six times more likely to be regular in attending treatment follow-up appointments compared to patients with poor medication adherence (OR 6.231 [95% CI 4.264–9.106]) (Table 3).

Similarly, for blood pressure control the results of binary regression analysis showed that treatment follow-up regularity, age, number of anti-hypertensive medications and medication adherence had significant association with blood pressure control. On the other hand, gender, body mass index (BMI), level of education, employment status, marital status, entitlement status, treatment duration and presence of co-morbidity had no significant association with controlled blood pressure. The results of binary regression analysis further revealed that odds of controlled blood pressure in patients who were regular in their treatment follow-up visits were 1.5 times higher than the patients who were irregular in their treatment follow-up visits (OR 1.561 [95% CI 1.102–2.211]). Similarly, the odds of controlled blood pressure were 1.08 times higher in males as compared to females (OR 1.08 [95% CI 0.753–1.543]), being 60 years of age and above (OR 1.638 [95% CI 1.168–2.297]). being unmarried/divorced/widowed (OR 1.257 [95% CI 0.757–2.087]), being university graduate (OR 1.229 [95% CI 0.730–2.068]), being normal weight (OR 1.05 [95% CI 0.743–1.482), being an officer (OR 1.462 [95% CI 0.661–3.236]), being entitled to free medical care (OR 1.210[95% CI 0.858–1.707]), more than 05 years of treatment duration (OR 1.246[95% CI 0.875–1.776]), having co-morbidity (OR 1.072 [95% CI 0.733–1.568]), being adherent to prescribed pharmacotherapy (OR 2.720[95% CI 1.890–3.915]). On the contrary the odds of controlled blood pressure decrease with every unit increase in no. of prescribed anti-hypertensive medications (OR 0.689[95% CI 0.538–0.882]). (Table 4).

**Table 4:** Factors predicting blood pressure control among included patients

Parameter	Uncontrolled B.P n (%)	Controlled B.P n (%)	OR	95% CI for OR lower–upper	P value
Gender					
Female	187 (53.9)	160 (46.1)	1		
Male	158 (50.2)	157 (49.8)	1.08	0.753–1.543	0.337
Age					
< 60 Years	243 (56.5)	187 (43.5)	1		
≥ 60 Years	102 (44.0)	130 (56.0)	1.638	1.168–2.297	0.004^*^
Marital status					
Married	305 (53.1)	269 (46.9)	1		
Unmarried/divorced/widowed	40 (45.5)	48 (54.5)	1.257	0.757–2.087	0.376
Education					
Uneducated	106 (56.7)	81 (43.3)	1		
Primary or below	73 (49.0)	76 (51.0)	1.431	0.904–2.265	0.126
Secondary and higher secondary	116 (51.3)	110 (48.7)	1.328	0.875–2.018	0.183
Graduation and above	50 (50.0)	50 (50.0)	1.229	0.730–2.068	0.437
BMI					
Obese	140 (53.6)	121 (46.4)	1		
Normal weight	205 (51.1)	196 (48.9)	1.05	0.743–1.482	0.782
Profession					
Retired/unemployed/house wives	216 (52.7)	194 (47.3)	1		
Officers	30 (47.6)	33 (52.4)	1.462	0.661–3.236	0.348
Clerical staff	25 (48.1)	27 (51.9)	1.320	0.640–2.721	0.453
Worker/laborer	65 (53.3)	57 (46.7)	1.276	0.746–2.185	0.374
Self employed	09 (60)	06 (40)	1.228	0.387–3.897	0.728
Entitlement status					
Non-entitled	228 (55.7)	181 (44.3)	1		
Entitled	117 (46.2)	136 (53.8)	1.210	0.858–1.707	0.277
Treatment duration					
≤ 5 years	247 (55.6)	197 (44.4)	1		
> 5 years	98 (45.0)	120 (55.0)	1.246	0.875–1.776	0.223
No. of medications					
1	149 (51.6)	140 (48.4)			
2	147 (50.2)	146 (49.8)	1		
3	48 (60.8)	31 (39.2)	0.689	0.538–0.882	0.003^*^
Co-morbidities					
No	265 (54.6)	220 (45.4)	1		
Yes	80 (45.2)	97 (54.8)	1.072	0.733–1.568	0.720
Medication adherence					
Non-Adherent	173 (67.8)	82 (32.2)	1		
Adherent	172 (42.3)	235 (57.7)	2.720	1.890–3.915	0.001^*^
Treatment follow-up regularity					
Irregular treatment follow-up	232 (59.8)	156 (40.2)	1		
Regular treatment follow-up	113 (41.2)	161 (58.8)	1.561	1.102–2.211	0.012^*^

Significant values are highlighted in bold

*P < 0.05

## Discussion

The aim of this study was to assess the regularity in attending treatment follow-up appointments, factors determining regularity in follow-up appointments and to evaluate the impact of regular treatment follow-up visits on treatment outcomes among patients with hypertension in Islamabad, Pakistan. Overall, attendance at treatment follow-up visits was poor. Furthermore, positive association between regular treatment follow-up and controlled blood pressure was observed among hypertension patients attending different healthcare settings. However, patients entitled to free medical care were more likely to attend treatment follow-up visits compared to the patients who had to pay from their own pocket.

Pakistan healthcare system consists of three tiers i.e. primary, secondary and tertiary healthcareand is run by both public and private sectors. Tertiary care hospitals are the teaching hospitals located in big cities of the country [23, 33]. Free healthcare coverage is available to only 27% of the population which mostly include government employees and members of the armed forces of Pakistan. They either receive free medicines and other healthcare services from the hospital or through reimbursement in case a medicine or a healthcare service is not available in the hospital. The remaining 73% population pay from their pockets for the healthcare services [34].

There is scarcity of research investigating the association between regularity in treatment follow-up appointments and blood pressure control not only in Pakistan but also in the South Asian region. In line with previous findings, we found a positive association between regular treatment follow-up and controlled blood pressure [8, 9, 15, 16, 35–37]. This positive association between regularity in treatment follow-up and controlled blood pressure could be due to the fact that the regular treatment follow-up visits provides an opportunity to healthcare providers to educate patients about their disease and drug therapy, optimize their therapy and to monitor treatment outcomes. There is a need to develop policies aiming at facilitating the patients in their treatment follow-up appointments to ensure better treatment outcomes.

Our results also suggest that entitlement to free medical care was significantly associated with regularity in treatment follow-up appointments. This finding is also consistent with previously reported studies [29, 38]. Unaffordability of healthcare services is the main issue in many lower-middle and low-income countries including Pakistan [39–41].. Health authorities should ensure that access to basic and specialised healthcare services is affordable for all the citizens.

A number of factors associated with regularity of treatment follow-up were identified in this study. It was observed that male patients were more regular in attending treatment follow-up appointments compared to the female patients. Gender differences in attending follow-up appointments have been reported for other disease conditions as well [42, 43]. However, studies conducted in the United Kingdom and Canada have reported that female patients were more likely to attend treatment follow-up visits compared to their male counterparts [14, 44]. The differences in the findings can be explained by the differences in cultural and social values. In Pakistan, females often need a male guardian to accompany them to the hospital and their attendance at. follow-up appointments depend on the availability of an accompanying person and this may lead to the less regularity in treatment follow-up among females. Female only clinics can potentially help overcome this barrier.

Age was found to be significantly associated with regularity in treatment follow-up visits. Previously, mixed results have been reported in literature for the association between age and regular attendance at follow-up appointments [14, 42–45]. Increased disease severity or the presence of other co-morbidities among old age patients may encourage elderly patients to regularly attend treatment follow-up appointments. However, some studies found negative correlation between age and regularity in treatment follow-up [46]. This negative correlation may be due to memory loss, non-availability of any caregiver or deteriorated health condition of the patient.

Our study found that patients with university level qualification were more likely to attend treatment follow-up visits. The results of our study are in line with previously conducted studies which also found a significant correlation between level of education and regularity in treatment follow-up visits [29, 47, 48]. Better disease knowledge and awareness about potential complications among university graduates may explain higher likelihood to attend treatment follow-up visits.

In line with the findings of a pervious study [46], we found significant positive correlation between the number of medications used and the regularity in treatment follow-up visits. A higher number of medications often means greater disease severity which could motivate patients to regularly attend their follow-up appointments. Similarly, in line with previous studies [14, 42], we found significant correlation between the presence of co-morbidity and regularity in treatment follow-up visits. Hypertension is often an asymptomatic disease so the patients do not feel need for regular treatment follow-up visits but the patients with any co-morbidity may perceive themselves sicker and seek medical help more frequently. Our study reported a very strong association between the medication adherence and regularity in treatment follow-up visits. This result is in line with previously conducted studies [49–51]. Similarly the result of our study suggests the significant correlation between the controlled blood pressure and regularity in treatment follow-up visits. Regularity in treatment follow-up visits increases the likelihood of controlled blood pressure in patients with hypertension. In every subgroup the patients who were regular in treatment follow-up visits had better rate of blood pressure control as compared to the patients who were regular in their treatment follow-up visits. These results are in line with the findings of previously conducted studies [8, 9, 14, 52]. This increased blood pressure control could be possibly because the regular follow-up visits provide healthcare practitioners an opportunity to adjust the treatment regimen, educate the patient and assess the patient’s adherence to prescribed pharmacotherapy, resulting in better treatment outcomes. Lastly high medication adherence was significantly associated with high rate of blood pressure control. The results of our study are self-explanatory and in line with a number of previous research studies than showed better treatment outcomes in patients with high medication adherence [3, 35, 53, 54].

### Implications for clinical practice

The findings of our study have quite significant practice and policy implications. There is a need to design and implement interventions to improve attendance at treatment follow-up appointments. In this study, we have identified several factors associated with regularity in treatment follow-up visits like gender, age, level of education, entitlement status, treatment duration, number of medications, presence of co-morbidity, medication adherence and controlled blood pressure. Hence any intervention targeting at any of these factors could be helpful in improving attendance during treatment follow-up appointments. These interventions need to be patient specific and patient related factors such as age, gender, health beliefs, culture, traditions and disease knowledge should be kept in mind when designing any intervention to improve attendance at treatment follow-up visits. The intervention to increase regularity in treatment follow-up visits may include, providing entitlement to free medical care, educational interventions for educating the patients regarding the benefits of regular treatment follow-up, motivational interviewing, family support, providing better services at healthcare settings, providing incentives to the patients on every visit for follow-up, sending reminders to the patients through phone calls, sms, and emails etc. [55–62]. There is a need to educate the patients who are at early stage of their disease to carefully follow the instructions provided by the healthcare professionals to halt further progression of their disease and its complications. Given that attendance at follow-up visits is associated with good BP control, healthcare professionals should emphasise the importance and purpose of attending follow-up visits during initial consultations. This will allow them to undertake required monitoring and optimise medicines to ensure best possible treatment outcomes for patients. In future studies, the distribution and causes of missed follow-up visits should also be explored in more detail.

### Study limitations and future perspective

There are few limitations to our study findings. This study was conducted only in one city of Pakistan, hence generalisation of findings to small towns and villages should be made with caution. We used self-reported questionnaires to access the regularity in treatment follow-up visits. Self-reported questionnaire has its own inherent disadvantages/limitation like patients ability to understand the questions in questionnaire and participant’s willingness to disclose his/her information. This may result in over or underestimation of the results. In addition, completing questionnaires involves recall of previous events which may lead to recall bias in old age patients or those who had been on treatment for many years.

## Conclusion

Regularity in treatment follow-up appointments was poor in patients with hypertension attending different healthcare settings in Islamabad, Pakistan. There is an association between blood pressure control and regularity in attending treatment follow-up appointments. On the basis of determining factors identified in our study, targeted interventions should be designed and implemented for better therapeutic outcomes.

## Data Availability

The data can be obtained from the corresponding author under reasonable request.

## Abbreviations

OR: Odds ratio
CI: Confidence interval
SD: Standard deviation
BP: Blood pressure
mmHg: Millimetre of mercury
MMAS-8: 8-Items morisky medication adherence scale
ESH: European society of hypertension
AHA: American heart association
NICE: National institute for health and care excellence
DM: Diabetes mellitus
CHD: Coronary heart disease
CHF: Congestive heart failure
HIV: Human immunodeficiency virus
BMI: Body mass index
SMS: Short message service
SPSS: Statistical package for social sciences
